# A New Lomax-G Family: Properties, Estimation and Applications

**DOI:** 10.3390/e27020125

**Published:** 2025-01-25

**Authors:** Hanan Baaqeel, Hibah Alnashshri, Lamya Baharith

**Affiliations:** 1 Department of Statistics, Faculty of Science, King Abdulaziz University, Jeddah 21589, Saudi Arabia; hbaageel@kau.edu.sa (H.B.); halnashere@kku.edu.sa (H.A.); 2 Department of Mathematics, King Khalid University, Abha 61421, Saudi Arabia

**Keywords:** new generalized family of distributions, Lomax distribution, Weibull distribution, Power–Lomax–Weibull, order statistics, Rényi entropy, maximum likelihood method, weighted least squares method, Cramér–von Mises method

## Abstract

Given the increasing number of phenomena that demand interpretation and investigation, developing new distributions and families of distributions has become increasingly essential. This article introduces a novel family of distributions based on the exponentiated reciprocal of the hazard rate function named the new Lomax-G family of distributions. We demonstrate the family’s flexibility to predict a wide range of lifetime events by deriving its cumulative and probability density functions. The new Lomax–Weibull distribution (NLW) is studied as a sub-model, with analytical and graphical evidence indicating its efficiency for reliability analysis and complex data modeling. The NLW density encompasses a variety of shapes, such as symmetrical, semi-symmetrical, right-skewed, left-skewed, and inverted J shapes. Furthermore, its hazard function exhibits a broad range of asymmetric forms. Five estimation techniques for determining the parameters of the proposed NLW distribution include the maximum likelihood, percentile, least squares, weighted least squares, and Cramér–von Mises methods. The performance of the estimators of the studied inferential methods is investigated through a comparative Monte Carlo simulation study and numerical demonstration. Additionally, the effectiveness of the NLW is validated by means of four real-world datasets. The results indicate that the NLW distribution provides a more accurate fit than several competing models.

## 1. Introduction

Statistical distributions effectively describe, analyze, and predict many global phenomena in applied fields such as medicine, engineering, economics, and insurance. Accurately analyzing and interpreting the data requires identifying the appropriate statistical distribution, as the validity of inferences and conclusions depends on the optimal distribution choice.

Classical and traditional distributions often fail to describe complex real-world data clearly and accurately. Therefore, it is essential to develop new techniques that generate families of statistical distributions that are more flexible and accurate in modeling diverse data types. Many statisticians are focused on devising methods to generate such families of distributions. One of the most notable of these families is the transformed-transformer (T-X) family, introduced by [1], a fully generalized family that is not restricted to any specific probability distribution. This family consists of two components used to create a new generalized family: the first component is the function representing the upper limit of integration, $W\left(F\left(x\right)\right)$, and the second component is the function to be integrated, where any probability distribution can be applied in either part randomly to form new families. Numerous studies have used this approach to develop new distributions and families of distributions. Examples include the Weibull–Pareto distribution introduced by [2], the exponentiated T-X distribution proposed by [3], and the Weibull–Normal distribution proposed by [4]. Furthermore, the generalized odd log-logistic family by [5], the Nadarajah Topp Leone-G family by [6], the odd Lomax-G family by [7], the modified T-X family by [8], and the exponential T-X family by [9]. More recently, [10] combined the Marshall–Olkin transformation with the T-X family to introduce the Marshall–Olkin Weibull family, and [11] proposed the generalized logarithmic–X family.

In a very recent study by [12], a new generalized family of distributions (NGF) was introduced using $W\left(F\left(x\right)\right)=m\left(x\right)$, where m(x) is known as the Mills ratio and is equivalent to the inverse of the hazard rate function [13]. The cumulative distribution function (CDF) and probability distribution function (PDF) of the NGF are as follows:

(1) $$F\left(x\right)=\int_{-\infty }^{m(x)}g\left(t\right)dt=G\left[m(x)\right]\;,\;x>0,$$

(2) $$f\left(x\right)=\left\{\frac{d}{dx}m(x)\right\}g\left\{m(x)\right\}.$$

Various applied disciplines can benefit from the increased flexibility of this method when modeling real-world data. Consequently, researchers have used it to create novel distributions. For example, [14] and colleagues introduced the survival-weighted Pareto distribution, while [15] proposed the reflected Pareto distribution.

The primary goal of this study is to propose a new family of distributions using the exponentiated reciprocal of the hazard rate function. It is important to note that the upper limit of the integral for creating the NGF family, given in (1), is m(x). Different upper limits of the integral can be defined to generate various families of distributions [2]. In this study, we define a new upper limit of the integral by introducing a shape parameter, c>0, such that the upper limit is set to $m^{c}\left(x\right)$. This will lead to new families of distributions with CDF and PDF defined as follows:

(3) $$F\left(x\right)=\int_{-\infty }^{m^{c}\left(x\right)}g\left(t\right)dt=G\left\{m^{c}\left(x\right)\right\},\;x>0,\;c>0,$$

(4) $$f\left(x\right)=\left\{\frac{d}{dx}m^{c}\left(x\right)\right\}g\left\{m^{c}\left(x\right)\right\},$$

where g(t) and G(t) represent the PDF and CDF of any probability distribution and $m^{c}\left(x\right)$ is a monotonically increasing function representing the exponentiated reciprocal of the hazard rate function for a baseline distribution.

**Figure 1:**
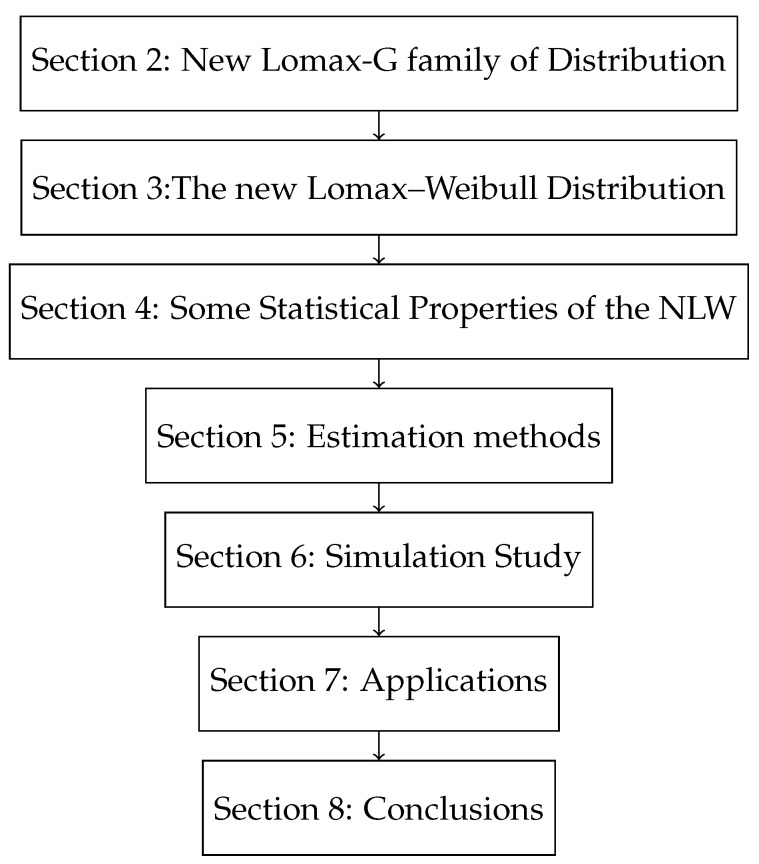
The organizational structure for the remaining sections of this article.

This article introduces the new generalized Lomax (NL-G) family based on the exponentiated reciprocal of the hazard rate function. We explore a specific member of the NL-G family, the new Lomax–Weibull distribution (NLW). The NLW distribution is highly effective for modeling real-world data across a wide range of applied disciplines, as it integrates features from both the Lomax family and the Weibull distribution. This combination offers wider applicability than the parent distributions, along with superior goodness of fit, improved estimation adaptability, and enhanced mathematical flexibility. Its versatility allows it to analyze diverse datasets in fields such as biology, computer science, marketing, medicine, economics, behavioral sciences, engineering, actuarial studies, environmental sciences, and lifetime analysis. This adaptability is evident in its ability to accommodate various shapes for both density and hazard functions. The remainder of this article is presented in Figure 1.

## 2. New Lomax-G Family of Distribution

Let x>0 be a random variable following the Lomax distribution with a shape parameter θ and a scale parameter λ. Then, the CDF is

(5) $$G\left(x\right)=1-{\left[1+\frac{x}{\lambda }\right]}^{-\theta },\;\theta ,\lambda >0.$$

The PDF corresponding to Equation (5) is

(6) $$g\left(x\right)=\frac{\theta }{\lambda }{\left[1+\frac{x}{\lambda }\right]}^{-(\theta +1)}\;.$$

Thus, $m^{c}\left(x\right)$ for the Lomax distribution can be defined as follows:

(7) $$m\left(x\right)=\frac{x+\lambda }{\theta }\;m^{c}\left(x\right)={\left(\frac{x+\lambda }{\theta }\right)}^{c}\;,x\geq 0.$$

Using the $m^{c}\left(x\right)$ of the Lomax distribution and Equations (3) and (4), we obtain the CDF and PDF of the NL-G family as follows:

(8) $$F\left(x\right)=\int_{0}^{{\left(\frac{x+\lambda }{\theta }\right)}^{c}}g\left(t\right)dt=G\left\{{\left(\frac{x+\lambda }{\theta }\right)}^{c}\right\},\;\theta >0,$$

(9) $$f\left(x\right)=\frac{c}{\theta }{\left(\frac{x+\lambda }{\theta }\right)}^{c-1}g\left[{\left(\frac{x+\lambda }{\theta }\right)}^{c}\right],$$

where λ is a location parameter.

## 3. The New Lomax–Weibull Distribution

In this section, we study a new special member of the NL-G family, the NLW, using the Weibull distribution as a baseline. The CDF of the NLW distribution can be obtained as

(10) $$F_{NLW}\left(x\right)=1-e^{-\alpha {\left(\frac{x+\lambda }{\theta }\right)}^{c\beta }},\;x\geq -\lambda .$$

Therefore, the PDF of the NLW model is obtained by differentiating Equation (10) as follows:

(11) $$\begin{array}{cc} f_{NLW}\left(x\right) & =\frac{c}{\theta }{\left(\frac{x+\lambda }{\theta }\right)}^{c-1}\alpha \beta {\left(\frac{x+\lambda }{\theta }\right)}^{c\beta -c}e^{-\alpha {\left(\frac{x+\lambda }{\theta }\right)}^{c\beta }}\\ \; & =\frac{\alpha \beta c}{\theta }{\left(\frac{x+\lambda }{\theta }\right)}^{c\beta -1}e^{-\alpha {\left(\frac{x+\lambda }{\theta }\right)}^{c\beta }}. \end{array}$$

The survival function, S(x), for the NLW is given by

(12) $$S\left(x\right)=1-F\left(x\right)=e^{-\alpha {\left(\frac{x+\lambda }{\theta }\right)}^{c\beta }},$$

and the hazard rate function, H(x), for the NLW is expressed as

(13) $$H\left(x\right)=\frac{\alpha \beta c}{\theta }{\left(\frac{x+\lambda }{\theta }\right)}^{c\beta -1}.$$

### 3.1. Graphical Presentations of the NLW

**Figure 2:**
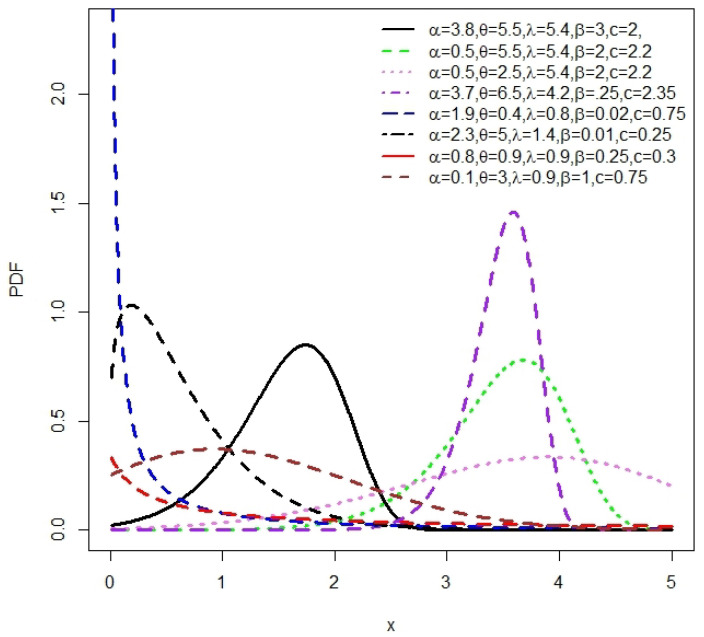
The NLW density plots for some values of α, β, λ, θ and *c*.

**Figure 3:**
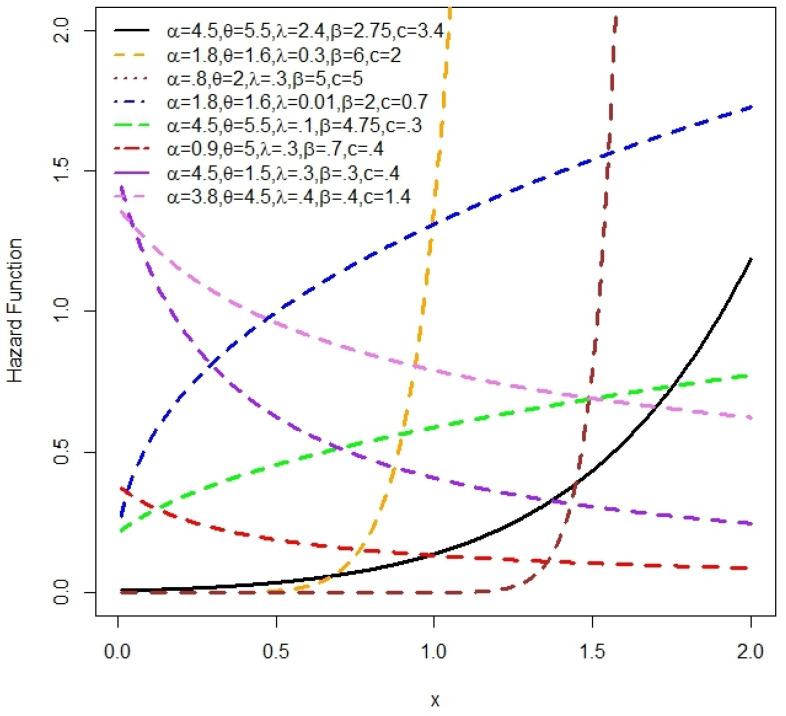
The NLW H(x) plots for some values of α, β, λ, θ and *c*.

The plots of the density function and H(x) for the NLW, based on some selected parameter values, are shown in Figure 2 and Figure 3, respectively.

Figure 2 illustrates that the density of the NLW can take on multiple forms, such as symmetrical, right-skewed, left-skewed, and inverted J-shapes. Additionally, the H(x) function of the NLW exhibits a diverse range of asymmetric shapes, including increasing, decreasing, J-shaped, and reversed J-shaped forms. These observations highlight the high flexibility of the NLW.

### 3.2. Special Cases of the NLW Distribution

1. The NLW distribution in (11) reduces to the exponential Lomax distribution with β=1andλ=θ.
2. The NLW distribution in (11) reduces to the Rayleigh distribution with $\alpha =\frac{1}{2},\;\beta =2\;\mathrm{and}\;c=1$.
3. For β=c=θ=1andy=x+λ,x∼NLW(α,λ,θ,β,c), Y has the exponential distribution.
4. For c=θ=1andy=x+λ,x∼NLW(α,λ,θ,β,c), Y has the Weibull distribution.
5. For $\beta =1\;,\alpha =\alpha^{c}\;\mathrm{and}\;y=\frac{x}{\lambda }+1,\;x∼NLW\left(\alpha ,\lambda ,\theta ,\beta ,c\right)$, Y has the Weibull distribution.

### 3.3. Useful Form of the NLW Density

A simple expansion of the PDF for the NLW distribution in (11) is provided below, using its series representation:

(14) $$f_{NLW}\left(x\right)=\frac{\alpha \beta c}{\theta }{\left(\frac{\lambda }{\theta }\right)}^{c\beta -1}{\left(1+\frac{x}{\lambda }\right)}^{c\beta -1}e^{-\alpha {\left(\frac{x+\lambda }{\theta }\right)}^{c\beta }}.$$

Consider the following binomial series representation:

(15) $${\left(1+z\right)}^{n}=\sum_{i=1}^{\infty }\left(\begin{array}{c} n\\ i \end{array}\right)z^{i}.$$

We have

$${\left(1+\frac{x}{\lambda }\right)}^{c\beta -1}=\sum_{i=1}^{\infty }\left(\begin{array}{c} c\beta -1\\ i \end{array}\right){\left(\frac{x}{\lambda }\right)}^{i}.$$

Therefore,

(16) $$f_{NLW}\left(x\right)=\frac{\alpha \beta c}{\theta^{c\beta }}\sum_{i=0}^{\infty }\left(\begin{array}{c} c\beta -1\\ i \end{array}\right)\lambda^{c\beta -i-1}x^{i}e^{-\alpha {\left(\frac{x+\lambda }{\theta }\right)}^{c\beta }}.$$

## 4. Some Statistical Properties of the NLW

### 4.1. Quantile Function

The *p*th quantile function, say, (0<p<1), of the NLW can be obtained as

(17) $$\begin{array}{cc} \; & x_{p}=\theta {\left[\frac{-1}{\alpha }ln\left(1-p\right)\right]}^{\frac{1}{c\beta }}-\lambda . \end{array}$$

As a result, by setting p=0.5 in Equation (17), we can derive the median of the NLW as follows:

(18) $$x_{0.5}=\theta {\left[\frac{-1}{\alpha }ln\left(0.5\right)\right]}^{\frac{1}{c\beta }}-\lambda .$$

The measures of deviation and skewness are calculated from the quantities using the following equations:

(19) $$S_{k}=\frac{x_{0.75}-2x_{0.5}+x_{0.25}}{x_{0.75}-x_{0.5}}.$$

and

(20) $$Kur=\frac{x_{0.875}-x_{0.625}-x_{0.375}+x_{0.125}}{x_{0.75}-x_{0.5}},$$

where $x_{\left(.\right)}$ is the quantile function.

### 4.2. Moments

If X∼NLW(α,β,λ,θ,c), then the $r^{th}$ moment of *X* can be obtained as

μr=Exr=∫−λ∞xrf(x)dx=αβcθcβ∑i=0∞cβ−1iλcβ−i−1∫−λ∞xi+re−α(x+λθ)cβdx.

Substituting $w=\alpha {\left(\frac{x+\lambda }{\theta }\right)}^{c\beta }$, we will have

μr=∑i=0∞cβ−1iλcβ+r−1θcβ−1∫0∞1−θλwα1cβi+rwα1cβ−1e−wdw.

Then, by employing the following series expansion

(21) $$\begin{array}{cc} {\left(1-z\right)}^{n} & =\sum_{j=0}^{n}{\left(-1\right)}^{j}\left(\begin{array}{c} n\\ j \end{array}\right)z^{j}. \end{array}$$

Therefore, the $r^{th}$ moment of the NLW can be written as

(22) μr=∑i=0∞∑j=0i+r−1i+j+ri+rjcβ−1iλcβ+r−j−1θcβ−j−1αj+1cβ−1Γj+1cβ.

The mean and variance of NLW are expressed as

(23) μ=E(x)=∑i=0∞∑j=0i+1−1i+j+1i+1jcβ−1iλcβ−jθcβ−j−1αj+1cβ−1Γj+1cβ.

(24) σ2=E(x2)−μ2=∑i=0∞∑j=0i+2−1i+j+2i+2jcβ−1iλcβ−j+1θcβ−j−1αj+1cβ−1Γj+1cβ−μ2.

### 4.3. Moment Generating Function

The moment generating function (MGF) of the random variable *x* is defined as

$$M_{x}\left(t\right)=\sum_{r=0}^{\infty }\frac{{\left(t\right)}^{r}}{r!}E\left(x^{r}\right).$$

Thus, we obtain the MGF as follows:

(25) Mx(t)=∑r=0∞∑i=1∞∑j=0r+i−1i+j+ri+rjcβ−1iλcβ+r−j−1θcβ−j−1αj+1cβ−1Γj+1cβtrr!.

### 4.4. Characteristic Function

The characteristic function is defined by

$$\phi_{x}\left(t\right)=E\left(e^{itx}\right)=\sum_{r=0}^{\infty }\frac{{\left(it\right)}^{r}}{r!}E\left(x^{r}\right).$$

Hence, the characteristic function of the NLW is given by

(26) ϕxt=∑r=0∞∑i=1∞∑j=0r+i−1i+j+ri+rjcβ−1iλcβ+r−j−1θcβ−j−1αj+1cβ−1Γj+1cβitrr!.

### 4.5. Probability Weighted Moment

The Probability Weighted Moment (PWM) for a random variable *X* that follows the NLW can be formally expressed as:

(27) $$E\left[x^{r}F{\left(x\right)}^{s}\right]=\int_{-\infty }^{\infty }x^{r}f\left(x\right){\left[F(x)\right]}^{s}dx.$$

By substituting Equations (10) and (11) into (27), the PWM for the NLW is derived in the following form:

(28) ExrF(x)s=∑i=1∞∑j=0s∑k=0r+i(−1)k+r+i+jr+iksjcβ−1iλcβ+r−k−1θcβ−k−1α1−k+1cβ(1+j)k+1cβΓk+1cβ.

### 4.6. Order Statistics

Let $x_{1}<x_{2}<x_{3}<\ldots <x_{n}$ represent a random sample of size *n* from the NLW, where $x_{1:n}$ denotes the $r^{th}$ order statistic. The PDF of $x_{1:n}$ can then be expressed as follows:

(29) fr:n(x)=f(x)β(r,n−r−1)∑v=0n−r(−1)vn−rvFv+r−1(x).

By substituting into Equations (11) and (12) into (29), and then applying the binomial series expansion in (21), we have

(30) fr:n(x)=αβcθβr,n−r−1∑v=0n−r∑k=0n(−1)kv+r−1kn−rvx+λθcβ−1×e−α(k+1)(x+λθ)cβ.

### 4.7. R’enyi Entropy

R’enyi entropy of order *u* is given by

(31) $$RE_{x}\left(u\right)=\frac{1}{1-u}log\left(\int_{-\infty }^{\infty }f^{u}\left(x\right)dx\right)\;u>0,u\neq 1.$$

Therefore, the Rényi entropy of the NLW distribution is given by

(32) $$\begin{array}{cc} RE_{x}\left(u\right) & =\frac{1}{1-u}\left[\left(u-1\right)log\left(\frac{\alpha \beta c}{\theta }\right)-klog\left(u\right)-\left(k-1\right)log\left(\alpha \right)+log\left(\Gamma \left(k\right)\right)\right],\; \end{array}$$

where $k=\frac{uc\beta -u+1}{c\beta }$.

### 4.8. Shannon Entropy

The Shannon entropy, $SE_{x}$, of X is defined as

(33) $$SE_{x}=-E\left[log\left(f(x)\right)\right].$$

Using the following sequence expansions, along with sequences (15) and (21),

(34) $$log\left(1+z\right)=\sum_{k=1}^{\infty }\frac{{\left(-1\right)}^{k}}{k}z^{k}.$$

Therefore, the Shannon entropy of the NLW is given by

(35) SEx=1−log(αβcθ)−(βc−1)×log(λθ)+∑k=1∞∑j=0cβ−1∑l=0k+j(−1)k+l−1kαl+1cβ−1cβ−1jk+jlλθcβ−l−1Γl+1cβ.

## 5. Estimation Methods

The parameters of the NLW distribution are estimated using five estimation methods: the maximum likelihood method (MLE), the percentile method (PE), the least squares method (LS), the weighted least squares method (WLS), and the Cramér–von Mises minimum distance method (CVM).

### 5.1. Maximum Likelihood Method

Let $x_{1},x_{2},x_{3},\ldots ,x_{n}$ represent a random sample of size *n* drawn from a NLW distribution. The log-likelihood function (*l*) for the parameter vector α,λ,θ,β,andc can be expressed as follows:

(36) $$\begin{array}{cc} l\left(\alpha ,\lambda ,\theta ,\beta ,c;x\right) & =nlog(\alpha )+nlog(\beta )+nlog(c)-nlog(\theta )\\ \; & +\left(c\beta -1\right)\sum_{i=1}^{n}log\left(\frac{x+\lambda }{\theta }\right)-\alpha \sum_{i=1}^{n}{\left(\frac{x+\lambda }{\theta }\right)}^{c\beta }. \end{array}$$

The MLE estimates of the parameters can then be derived by differentiating (36) with respect to each parameter and setting the resulting equations to zero, yielding the following:

(37) $$\frac{\partial l}{\partial \alpha }=\frac{n}{\alpha }-\sum_{i=1}^{n}{\left(\frac{x+\lambda }{\theta }\right)}^{c\beta }.$$

(38) $$\frac{\partial l}{\partial \lambda }=\frac{c\beta -1}{n\lambda +\sum_{i=1}^{n}\left(x\right)}-\frac{\alpha \beta c}{\theta }\sum_{i=1}^{n}{\left(\frac{x+\lambda }{\theta }\right)}^{c\beta -1}.$$

(39) $$\frac{\partial l}{\partial \theta }=\frac{n}{\theta }\left(c\beta -2\right)+\frac{\alpha \beta c}{\theta }\sum_{i=1}^{n}{\left(\frac{x+\lambda }{\theta }\right)}^{c\beta }.$$

(40) $$\frac{\partial l}{\partial \beta }=\frac{n}{\beta }+c\sum_{i=1}^{n}log\left(\frac{x+\lambda }{\theta }\right)-\alpha c\sum_{i=1}^{n}{\left(\frac{x+\lambda }{\theta }\right)}^{c\beta }log\left(\frac{x+\lambda }{\theta }\right).$$

(41) $$\frac{\partial l}{\partial c}=\frac{n}{c}+\beta \sum_{i=1}^{n}log\left(\frac{x+\lambda }{\theta }\right)-\alpha \beta \sum_{i=1}^{n}{\left(\frac{x+\lambda }{\theta }\right)}^{c\beta }log\left(\frac{x+\lambda }{\theta }\right).$$

The maximum error for each parameter can then be determined by solving the system of Equations (37)–(41) using iterative optimization techniques. Consequently, these optimization methods can be implemented in any statistical software (e.g., R packages).

### 5.2. Percentiles Method

Consider a random sample $x_{1},x_{2},x_{3},\ldots ,x_{n}$ of size *n* drawn from an NLW. The percentile estimation (PE) method is formulated as

(42) $$PE\left(\alpha ,\lambda ,\theta ,\beta ,c\right)=\sum_{i=1}^{n}\left[x_{i}-\left(\theta {\left[\frac{-1}{\alpha }ln\left(1-p\right)\right]}^{\frac{1}{c\beta }}-\lambda \right)\right].$$

where $p_{i}=\frac{i}{n+1}\;\mathrm{and}\;i=1,2,3,\ldots ,n.$

To estimate the unknown parameters α,λ,θ,β,andc, the PE method minimizes the squared deviations between the observed sample order statistics and the expected values derived from the specified percentiles.

### 5.3. Ordinary and Weighted Least Squares Estimators

Let $x_{1},x_{2},x_{3},\ldots ,x_{n}$ be a random sample of size n drawn from NLW. The ordinary least squares estimator (LSE) method can be expressed as follows:

(43) $$LSE\left(\alpha ,\lambda ,\theta ,\beta ,c\right)=\sum_{i=1}^{n}{\left[F_{NLW}\left(x_{i}|\alpha ,\lambda ,\theta ,\beta ,c\right)-\left(\frac{i}{n+1}\right)\right]}^{2},\;i=1,2,\ldots ,n.$$

by minimizing the sum of squares errors for the unknown parameters. Thus, the ordinary least squares (LSE) estimator for the unknown parameters α,λ,θ,β,andc is obtained by minimizing the following quantity:

(44) $$LSE\left(\alpha ,\lambda ,\theta ,\beta ,c\right)=\sum_{i=1}^{n}{\left[1-e^{-\alpha {\left(\frac{x_{i}+\lambda }{\theta }\right)}^{c\beta }}-\left(\frac{i}{n+1}\right)\right]}^{2},\;i=1,2,\ldots ,n.$$

and the weighted least squares estimators (WLSs) of α,λ,θ,β,andc can be obtained by minimizing the following expression:

(45) $$WLS\left(\alpha ,\lambda ,\theta ,\beta ,c\right)=\sum_{i=1}^{n}\frac{{\left(n+1\right)}^{2}\left(n+2\right)}{i\left(n-i+1\right)}{\left[1-e^{-\alpha {\left(\frac{x_{i}+\lambda }{\theta }\right)}^{c\beta }}-\left(\frac{i}{n+1}\right)\right]}^{2}.$$

### 5.4. Ke Cramer–von Mises Minimum Distance Method

The Ke Cramer–von Mises minimum distance estimator (CVM) method relies on the discrepancy between the estimated CDF and the empirical CDF. CVM estimators are derived by minimizing this discrepancy.

(46) $$CVM=\frac{1}{12n}+\sum_{i=1}^{n}{\left[F\left(x_{i}\right)-\frac{2i-1}{2n}\right]}^{2},$$

By substituting Equation (10) into (46), the CVM estimator for α, λ, θ, β, and *c* can be obtained by minimizing the following expression:

(47) $$CVM=\frac{1}{12n}+\sum_{i=1}^{n}{\left[1-e^{-\alpha {\left(\frac{x_{i}+\lambda }{\theta }\right)}^{c\beta }}-\frac{2i-1}{2n}\right]}^{2}.$$

## 6. Simulation Study

This section presents numerical results from a Monte Carlo simulation to compare estimation methods, specifically MLE, PE, LSE, WLS, and CVM, in assessing the performance of these methods with respect to the NLW parameters.

- Data are generated from the NLW distribution as described in Equation (17), with p∼uniform(0,1).
- We examine multiple sample sizes (20, 50, 100, 200, 300, and 500) from the NLW, each under 1000 repetitions.
- Four distinct sets of parameter values are defined as follows:
  - Set I: (α=1.65,λ=0.055,θ=0.59,β=1.98,c=2.5).
  - Set II: (α=1.9,λ=0.07,θ=2.5,β=1.2,c=1.9).
  - Set III: (α=1.4,λ=0.117,θ=1.27,β=1.2,c=1.45).
  - Set IV: (α=26.4,λ=11.7,θ=12.77,β=15.2,c=33.45).

Using the “optim” function in the R statistical software (v4.3.2; R Core Team 2023) [16], we calculated parameter estimates ($\hat{\varphi }$) as well as the mean bias (Bias) and the mean squared error (MSE) defined as follows:

$$MSE\left(\hat{\varphi }\right)=\frac{1}{n}\sum_{i=1}^{n}{\left({\hat{\varphi }}_{i}-\varphi \right)}^{2},$$

where φ is the true value of the parameter, $\hat{\varphi }$ is the estimate of the parameter, and *n* is the sample size.

$$Bias\left(\hat{\varphi }\right)=\frac{1}{n}\sum_{i=1}^{n}\left({\hat{\varphi }}_{i}-\varphi \right).$$

The simulation results are presented in Table 1, Table 2, Table 3 and Table 4.

**Figure 4:**
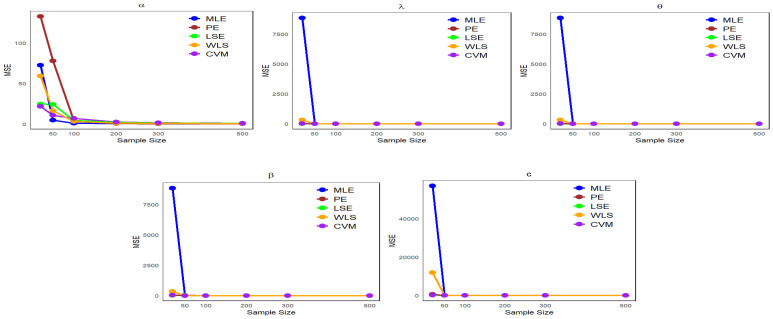
Comparison of estimation methods based on MSE across different sample sizes for Table 1.

**Figure 5:**
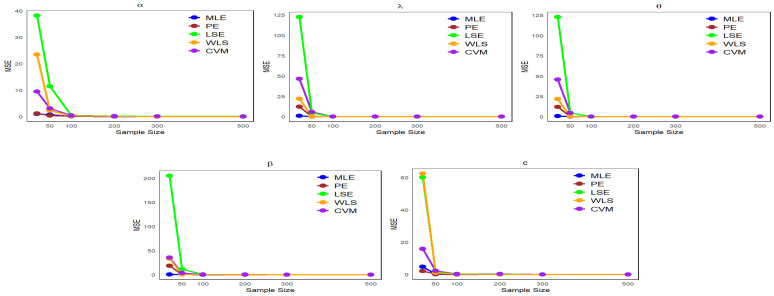
Comparison of estimation methods based on MSE Across different sample sizes for Table 2.

**Figure 6:**
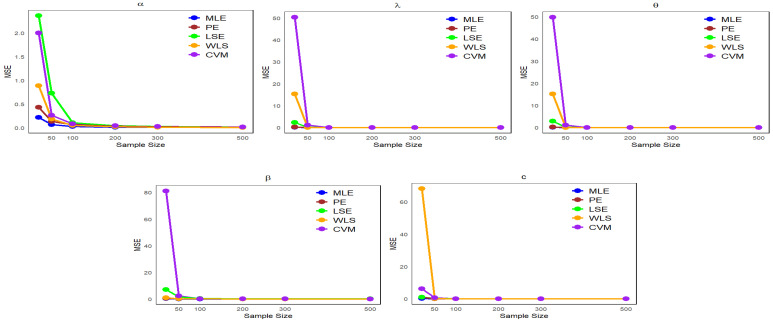
Comparison of estimation methods based on MSE Across different sample sizes for Table 3.

**Figure 7:**
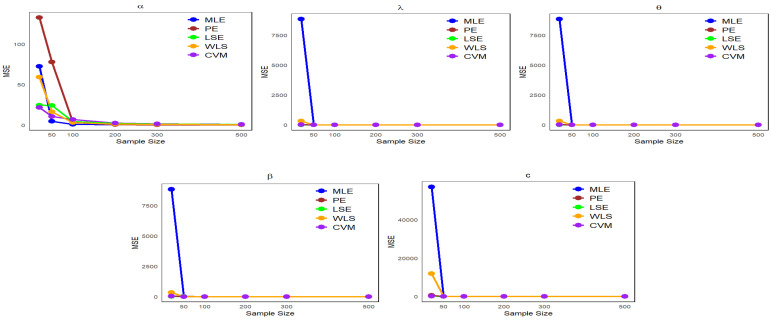
Comparison of estimation methods based on MSE across different sample sizes for Table 4.

It can be observed from Table 1, Table 2, Table 3 and Table 4 that the mean estimates of the NLW parameters approach the true values of the parameters (φ) as the sample size increases, reflecting a clear improvement in the accuracy of the estimates with increasing data. In addition, Figure 4, Figure 5, Figure 6 and Figure 7 show a comparison of estimation methods based on MSEs at different sample sizes. Consequently, the ML and WLS methods are more accurate than other methods in terms of accuracy and consistency when predicting unknown parameters of the NLW distribution. All analyses were performed using R Statistical Software (v4.3.2; R Core Team 2023) [16].

## 7. Applications

The NLW model has been applied to four real datasets to demonstrate its effectiveness in modeling real data compared to other distributions.

**Failure Time Data:**
The first dataset, obtained from [17], represents 84 recorded failure times of aircraft windshields, described as follows: 0.040, 1.866, 2.385, 3.443, 0.301, 1.876, 2.481, 3.467, 0.309, 1.899, 2.610, 3.478, 0.557, 1.911, 2.625, 3.578, 0.943, 1.912, 2.632, 3.595, 1.070, 1.914, 2.646, 3.699, 1.124, 1.981, 2.661, 3.779, 1.248, 2.010, 2.688, 3.924, 1.281, 2.038, 2.823, 4.035, 1.281, 2.085, 2.890, 4.121, 1.303, 2.089, 2.902, 4.167, 1.432, 2.097, 2.934, 4.240, 1.480, 2.135, 2.962, 4.255, 1.505, 2.154, 2.964, 4.278, 1.506, 2.190, 3.000, 4.305, 1.568, 2.194, 3.103, 4.376, 1.615, 2.223, 3.114, 4.449, 1.619, 2.224, 3.117, 4.485, 1.652, 2.229, 3.166, 4.570, 1.652, 2.300, 3.344, 4.602, 1.757, 2.324, 3.376, 4.663.
**Gauge Lengths of 10 mm Data:**
The second dataset was obtained from [18] and consists of 63 observations: 1.901, 2.132, 2.203, 2.228, 2.257, 2.350, 2.361, 2.396, 2.397, 2.445, 2.454, 2.474, 2.518, 2.522, 2.525, 2.532, 2.575, 2.614, 2.616, 2.618, 2.624, 2.659, 2.675, 2.738, 2.740, 2.856, 2.917, 2.928, 2.937, 2.937, 2.977, 2.996, 3.030, 3.125, 3.139, 3.145, 3.220, 3.223, 3.235, 3.243, 3.264, 3.272, 3.294, 3.332, 3.346, 3.377, 3.408, 3.435, 3.493, 3.501, 3.537, 3.554, 3.562, 3.628, 3.852, 3.871, 3.886, 3.971, 4.024, 4.027, 4.225, 4.395, 5.020.
**Strength Data:**
The third dataset, sourced from [19], includes 63 observations: 0.55, 0.74, 0.77, 0.81, 0.84, 1.24, 0.93, 1.04, 1.11, 1.13, 1.30, 1.25, 1.27, 1.28, 1.29, 1.48, 1.36, 1.39, 1.42, 1.48, 1.51, 1.49, 1.49, 1.50, 1.50, 1.55, 1.52, 1.53, 1.54, 1.55, 1.61, 1.58, 1.59, 1.60, 1.61, 1.63, 1.61, 1.61, 1.62, 1.62, 1.67, 1.64, 1.66, 1.66, 1.66, 1.70, 1.68, 1.68, 1.69, 1.70, 1.78, 1.73, 1.76, 1.76, 1.77, 1.89, 1.81, 1.82, 1.84, 1.84, 2.00, 2.01, 2.24.
**Student Grades in Mathematics Data:**
The fourth dataset represents the mathematical scores of 48 students in the slow pace program in the year 2013, sourced from [20]. The data are as follows: 29, 25, 50, 15, 13, 27, 15, 18, 7, 7, 8, 19, 12, 18, 5, 21, 15, 86, 21, 15, 14, 39, 15, 14, 70, 44, 6, 23, 58, 19, 50, 23, 11, 6, 34, 18, 28, 34, 12, 37, 4, 60, 20, 23, 40, 65, 19, and 31.

The suitability of the NLW model for the four datasets is evaluated by comparing its fit with the following distributions:

- Lomax distribution with the CDF function given in (5).
- Truncated Weibull power Lomax distribution (TWPL) by [21]
  $$F\left(x\right)={\left(1-e^{-1}\right)}^{-1}\left(1-e^{-{\left(1-{\left(1+\frac{x^{\beta }}{\gamma }\right)}^{-\alpha }\right)}^{\lambda }}\right),\;\alpha ,\beta ,\gamma ,\lambda ,x>0.$$
- Odd Lomax inverse Weibull distribution (OILW) by [22]
  $$F\left(x\right)=1-\beta^{\alpha }{\left[\beta +\frac{e^{-{\left(\frac{\theta }{x}\right)}^{\lambda }}}{1-e^{-{\left(\frac{\theta }{x}\right)}^{\lambda }}}\right]}^{-\alpha },\;\alpha ,\beta ,\lambda ,\theta ,x>0.$$
- The exponentiated generalized modified Weibull distribution (EGMW) by [23]
  $$F\left(x\right)={\left[1-e^{-\alpha \left(\theta x+\mu x^{\lambda }\right)}\right]}^{\beta },\;\alpha ,\theta ,\mu ,\lambda ,\beta >0.$$

ML estimates, standard error (SE), and the length of confidence intervals of the NLW model for the four datasets are presented in Table 5, offering a deeper statistical understanding of the data.

Additionally, the results in Table 6, Table 7, Table 8 and Table 9 show that the NLW model consistently achieves the lowest goodness-of-fit (GoF) criteria, including the Akaike Information Criterion (AIC), the Bayesian Information Criterion (BIC), Consistent Akaike Information Criterion (CAIC), the Hannan–Quinn Information Criterion (HQIC), the Kolmogorov–Smirnov (K-S) test, and the Anderson-Darling (A-D) test, indicating it provides the best fit to the data compared to other competing distributions. Furthermore, the distributions were compared using the *p*-values obtained from the K-S test, which revealed that the NLW model achieved the highest *p*-value, further supporting its superior fit. These results underscore the robustness of the NLW model in accurately capturing the characteristics of the data.

**Figure 8:**
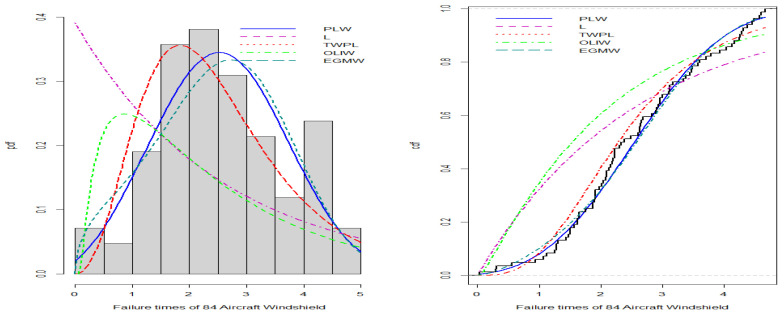
Estimated PDF and CDF for failure time data.

**Figure 9:**
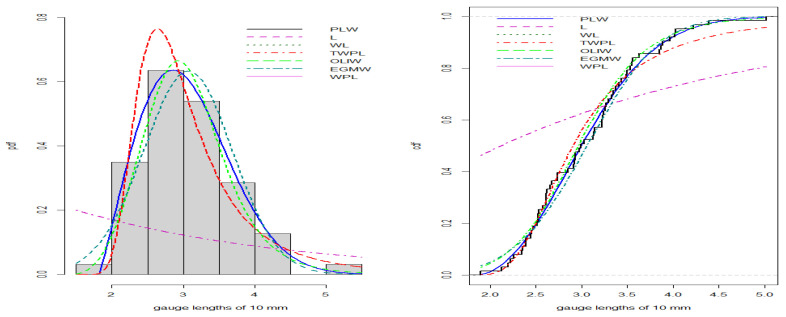
Estimated PDF and CDF for gauge data.

**Figure 10:**
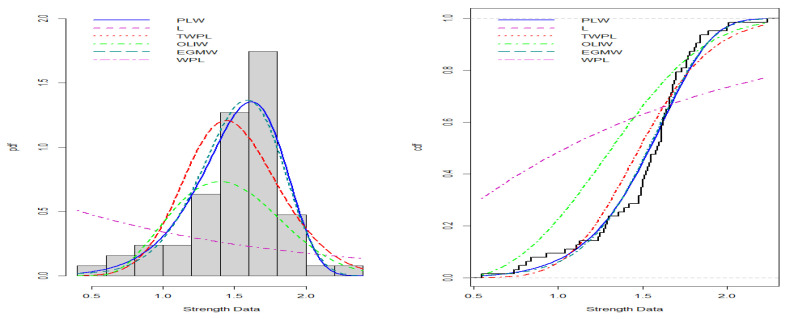
Estimated PDF and CDF for the strength data.

**Figure 11:**
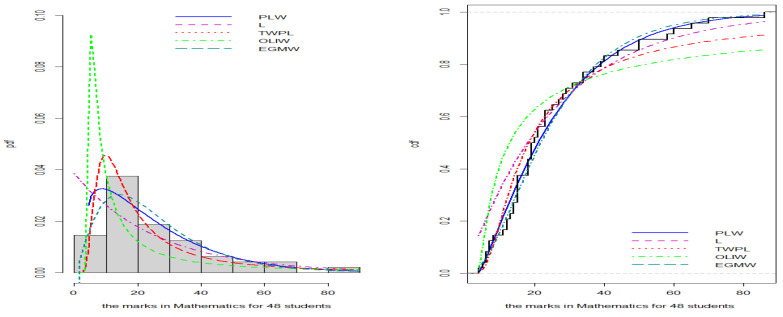
Estimated PDF and CDF for the student grades data.

Figure 8, Figure 9, Figure 10 and Figure 11 illustrate the PDFs and CDFs for all datasets, demonstrating that the NLW model fits the data exceptionally well, effectively capturing the skewness of the four data compared to other distributions.

## 8. Conclusions

This paper introduces a novel family based on the exponentiated reciprocal of the hazard rate function called the new Lomax generalized family (NL-G). A notable submodel within this family is the new Lomax–Weibull (NLW) distribution, which exhibits remarkable adaptability and is capable of fitting diverse datasets. The NLW’s density function is highly versatile, taking on various forms, including symmetric, semi-symmetric, inverted J-shape, right-skewed, and left-skewed shapes. Additionally, its hazard function exhibits a broad range of asymmetric shapes, such as increasing, decreasing, J-shaped, and inverted J-shaped forms, demonstrating a great deal of flexibility for real-world applications. The article outlines several key statistical properties of the NLW, such as quantiles, medians, moments, characteristic function, order statistics, and Rényi and Shannon entropies. We perform parameter estimation using the MLE, PE, LSE, WLS, and CVM methods, and their performance is evaluated through Monte Carlo simulation. The MLE and WLS methods demonstrated superior performance and consistency in parameter prediction. The practicality of the NLW model is showcased through its application to four real-world datasets, where it outperforms other established distributions, highlighting its exceptional ability to model and interpret complex data patterns across various domains.

## Figures and Tables

**Table 1:** Simulation study at α = 1.65, λ = 0.055, θ = 0.59, β = 1.98 and *c* = 2.5.

Set I:			MLE			PE			LSE			WLS			CVM	
		Est	Bias	MSE	Est	Bias	MSE	Est	Bias	MSE	Est	Bias	MSE	Est	Bias	MSE
	α	2.8325	1.1825	72.398	2.6589	1.0089	132.64	2.3626	0.7126	24.436	2.6864	1.0364	59.301	2.5847	0.9347	21.647
	λ	5.1111	5.0561	8850.1	1.0389	0.9839	39.867	0.7355	0.6805	12.033	1.2694	1.2144	336.58	0.7673	0.7123	32.162
n = 20	θ	5.6321	5.0421	8849.2	1.5375	0.9475	39.972	1.2582	0.6682	12.088	1.7878	1.1978	336.61	1.2956	0.7056	32.190
	β	8.4348	6.4548	17,322	4.7218	2.7418	404.88	3.8820	1.9020	156.54	3.8012	1.8212	88.329	4.7230	2.7430	558.78
	*c*	17.342	14.842	57,031	4.9615	2.4615	528.04	4.4392	1.9392	163.37	8.2053	5.7053	11,922	4.2164	1.7164	115.83
	α	1.9553	0.3053	4.5297	2.4883	0.8383	77.746	2.3747	0.7247	24.084	2.1694	0.5194	16.214	2.2274	0.5774	10.746
	λ	0.2441	0.1891	1.3782	0.5950	0.5400	13.540	0.4685	0.4135	4.4953	0.3447	0.2897	1.9793	0.3371	0.2821	1.9451
n = 50	θ	0.7697	0.1797	1.3349	1.1145	0.5245	13.632	1.0008	0.4108	4.5406	0.8682	0.2782	2.0244	0.8665	0.2765	1.9879
	β	2.6559	0.6759	20.546	3.9032	1.9232	185.936	3.2885	1.3085	55.768	2.8668	0.8868	43.128	3.0731	1.0931	32.324
	*c*	3.1397	0.6397	33.975	3.5148	1.0148	46.820	3.7666	1.2666	61.384	3.5547	1.0547	35.8343	3.5488	1.0488	36.623
	α	1.7792	0.1292	0.9413	1.8632	0.2132	3.2869	2.0475	0.3975	4.3204	1.9690	0.3190	3.1240	2.2048	0.5548	6.9803
	λ	0.0942	0.0392	0.0736	0.2019	0.1469	1.4697	0.2056	0.1506	0.3953	0.1863	0.1313	0.4331	0.2465	0.1915	1.4830
n = 100	θ	0.6237	0.0337	0.0591	0.7299	0.1399	1.4916	0.7389	0.1489	0.4042	0.7174	0.1274	0.4330	0.7825	0.1925	1.5185
	β	2.2200	0.2400	2.5263	2.4232	0.4432	15.3716	2.5633	0.5833	10.510	2.3945	0.4145	4.0481	2.5336	0.5536	8.0548
	*c*	2.6651	0.1651	1.2119	2.8840	0.3840	10.539	3.1386	0.6386	11.385	3.0895	0.5895	11.886	3.6041	1.1041	28.298
	α	1.7746	0.1246	0.3944	1.6937	0.0437	0.5465	1.8919	0.2419	2.1012	1.7869	0.1369	1.1100	1.9395	0.2895	2.4003
	λ	0.0671	0.0121	0.0154	0.1021	0.0471	0.0224	0.1483	0.0933	0.1531	0.1062	0.0512	0.0786	0.1371	0.0821	0.1316
n = 200	θ	0.6044	0.0144	0.0124	0.6317	0.0417	0.0192	0.6827	0.0927	0.1577	0.6372	0.0472	0.0808	0.6720	0.0820	0.1378
	β	2.0513	0.0713	0.3637	2.0980	0.1180	0.8957	2.2493	0.2693	2.8724	2.1583	0.1783	1.2778	2.2319	0.2519	1.8202
	*c*	2.5894	0.0894	0.5197	2.7698	0.2698	1.1236	3.0235	0.5235	5.7526	2.8152	0.3152	3.4335	3.1113	0.6113	9.9644
	α	1.7939	0.1439	0.2853	1.6876	0.0376	0.3301	1.8286	0.1786	1.2810	1.7377	0.0877	0.7493	1.8746	0.2246	1.5704
	λ	0.0585	0.0035	0.0084	0.0830	0.0280	0.0114	0.0996	0.0446	0.0371	0.0850	0.0300	0.0185	0.1161	0.0611	0.0741
n = 300	θ	0.5997	0.0097	0.0073	0.6152	0.0252	0.0102	0.6331	0.0431	0.0385	0.6163	0.0263	0.0182	0.6508	0.0608	0.0797
	β	2.0125	0.0325	0.2397	2.0160	0.0360	0.3186	2.1325	0.1525	0.9961	2.1486	0.1686	0.7590	2.2211	0.2411	1.4580
	*c*	2.5625	0.0625	0.3312	2.7001	0.2001	0.7051	2.8599	0.3599	2.5825	2.6639	0.1639	1.0250	2.8540	0.3540	3.5211
	α	1.7493	0.0993	0.1477	1.6977	0.0477	0.2407	1.8173	0.1673	0.9646	1.7547	0.1047	0.3717	1.8012	0.1512	0.7829
	λ	0.0549	0.0001	0.0043	0.0729	0.0179	0.0058	0.0848	0.0298	0.0200	0.0748	0.0198	0.0095	0.0906	0.0356	0.0219
n = 500	θ	0.5947	0.0047	0.0042	0.6081	0.0181	0.0063	0.6214	0.0314	0.0223	0.6113	0.0213	0.0111	0.6259	0.0359	0.0256
	β	1.9680	0.0120	0.1007	1.9842	0.0042	0.1723	2.0938	0.1138	0.8366	2.0574	0.0774	0.3413	2.0809	0.1009	0.5584
	*c*	2.5651	0.0651	0.1959	2.6496	0.1496	0.3651	2.7682	0.2682	1.4894	2.6361	0.1361	0.6002	2.7961	0.2961	1.5130

**Table 2:** Simulation study at α = 1.9, λ = 0.07, θ = 2.5, β = 1.2 and *c* = 1.9.

Set II:			MLE			PE			LSE			WLS			CVM	
		Est	Bias	MSE	Est	Bias	MSE	Est	Bias	MSE	Est	Bias	MSE	Est	Bias	MSE
	α	2.1252	0.2252	1.0045	1.7955	0.1045	1.2215	2.2165	0.3165	38.1752	2.0635	0.1635	23.4578	1.9887	0.0887	9.5050
	λ	0.0987	0.0287	1.1627	0.5046	0.4346	12.358	1.6284	1.5584	122.41	0.9587	0.8887	21.980	1.1731	1.1031	46.391
n = 20	θ	2.5924	0.0924	0.7104	2.8532	0.3532	11.936	3.8656	1.3656	122.62	3.2268	0.7268	21.743	3.3836	0.8836	45.636
	β	1.2414	0.0414	0.4173	1.5393	0.3393	18.189	2.8180	1.6180	204.99	2.0297	0.8297	33.370	2.0745	0.8745	35.206
	*c*	2.1433	0.2433	4.7736	2.1248	0.2248	2.0812	2.6289	0.7289	59.894	2.6107	0.7107	62.280	2.5608	0.6608	15.909
	α	2.1307	0.2307	0.7892	1.9076	0.0076	0.3990	1.9119	0.0119	11.4561	1.8608	0.0392	2.2713	1.9803	0.0803	3.1521
	λ	0.0065	0.0635	0.0832	0.1508	0.0808	0.1940	0.4593	0.3893	4.4835	0.2731	0.2031	0.5199	0.3960	0.3260	5.5061
n = 50	θ	2.5415	0.0415	0.0649	2.6125	0.1125	0.1047	2.7698	0.2698	4.4822	2.6272	0.1272	0.4337	2.7516	0.2516	5.4158
	β	1.2106	0.0106	0.1642	1.2528	0.0528	0.2828	1.5703	0.3703	11.2381	1.3601	0.1601	0.4883	1.4959	0.2959	3.5417
	*c*	1.9035	0.0035	0.2328	1.9896	0.0896	0.3995	2.1333	0.2333	1.5769	2.0581	0.1581	1.0788	2.1096	0.2096	2.3547
	α	2.0788	0.1788	0.1316	1.9657	0.0657	0.1995	1.8549	0.0451	0.4114	1.9071	0.0071	0.3292	1.9115	0.0115	0.4935
	λ	0.0211	0.0489	0.0265	0.0808	0.0108	0.0738	0.2438	0.1738	0.2795	0.1624	0.0924	0.0945	0.1821	0.1121	0.1618
n = 100	θ	2.5468	0.0468	0.0200	2.5660	0.0660	0.0444	2.6163	0.1163	0.2021	2.5788	0.0788	0.0859	2.5791	0.0791	0.1117
	β	1.1956	0.0044	0.0479	1.1971	0.0029	0.0970	1.3539	0.1539	0.4883	1.2887	0.0887	0.1772	1.3357	0.1357	0.4655
	*c*	1.8834	0.0166	0.0563	1.9369	0.0369	0.1656	2.0044	0.1044	0.3875	1.9593	0.0593	0.2628	1.9822	0.0822	0.3541
	α	2.0424	0.1424	0.0618	1.9764	0.0764	0.0981	1.9197	0.0197	0.1855	1.9314	0.0314	0.1041	1.9408	0.0408	0.2055
	λ	0.0362	0.0338	0.0118	0.0713	0.0013	0.0285	0.1559	0.0859	0.0747	0.1151	0.0451	0.0304	0.1499	0.0799	0.0726
n = 200	θ	2.5430	0.0430	0.0106	2.5541	0.0541	0.0229	2.5829	0.0829	0.0807	2.5576	0.0576	0.0320	2.5777	0.0777	0.0798
	β	1.1797	0.0203	0.0150	1.1957	0.0043	0.0393	1.2819	0.0819	0.1564	1.2524	0.0524	0.0969	1.2743	0.0743	0.1435
	*c*	1.9053	0.0053	0.0298	1.9218	0.0218	0.0787	1.9495	0.0495	0.2030	1.9231	0.0231	0.0920	1.9752	0.0752	0.2719
	α	2.0166	0.1166	0.0380	1.9730	0.0730	0.0558	1.9596	0.0596	0.1138	1.9487	0.0487	0.0529	1.9567	0.0567	0.1642
	λ	0.0434	0.0266	0.0069	0.0610	0.0090	0.0168	0.1309	0.0609	0.0433	0.1002	0.0302	0.0177	0.1272	0.0572	0.0400
n = 300	θ	2.5379	0.0379	0.0078	2.5436	0.0436	0.0156	2.5871	0.0871	0.0524	2.5544	0.0544	0.0205	2.5721	0.0721	0.0601
	β	1.1803	0.0197	0.0089	1.1883	0.0117	0.0261	1.2590	0.0590	0.0888	1.2213	0.0213	0.0329	1.2619	0.0619	0.0853
	*c*	1.9060	0.0060	0.0150	1.9117	0.0117	0.0440	1.9288	0.0288	0.1138	1.9270	0.0270	0.0504	1.9278	0.0278	0.1087
	α	2.0036	0.1036	0.0245	1.9669	0.0669	0.0336	1.9546	0.0546	0.0943	1.9447	0.0447	0.0283	1.9516	0.0516	0.0640
	λ	0.0491	0.0209	0.0039	0.0630	0.0070	0.0104	0.1132	0.0432	0.0238	0.0909	0.0209	0.0099	0.1044	0.0344	0.0207
n = 500	θ	2.5361	0.0361	0.0048	2.5364	0.0364	0.0123	2.5675	0.0675	0.0411	2.5441	0.0441	0.0117	2.5555	0.0555	0.0283
	β	1.1843	0.0157	0.0048	1.1931	0.0069	0.0188	1.2515	0.0515	0.0757	1.2181	0.0181	0.0181	1.2381	0.0381	0.0451
	*c*	1.9033	0.0033	0.0068	1.9022	0.0022	0.0249	1.9078	0.0078	0.0626	1.9130	0.0130	0.0310	1.9124	0.0124	0.0653

**Table 3:** Simulation study at α=1.4,λ=0.117,θ=1.27,β=1.2 and c=1.45.

Set III:			MLE			PE			LSE			WLS			CVM	
		Est	Bias	MSE	Est	Bias	MSE	Est	Bias	MSE	Est	Bias	MSE	Est	Bias	MSE
	α	1.5814	0.1814	0.2225	1.3867	0.0133	0.4314	1.3496	0.0504	2.9444	1.2654	0.1346	0.8901	1.3059	0.0941	2.0009
	λ	0.0746	0.0424	0.1545	0.2214	0.1044	0.3504	0.4121	0.2951	2.4036	0.5367	0.4197	15.3767	0.6711	0.5541	50.310
n = 20	θ	1.2910	0.0210	0.1152	1.3797	0.1097	0.2618	1.4575	0.1875	2.3614	1.5732	0.3032	15.1566	1.6915	0.4215	49.765
	β	1.1544	0.0456	0.2761	1.2256	0.0256	0.5083	1.5165	0.3165	7.0105	1.4214	0.2214	0.9747	1.9180	0.7180	81.094
	*c*	1.4935	0.0435	0.1782	1.6275	0.1775	0.9548	1.6856	0.2356	0.9860	2.1021	0.6521	68.0949	1.8635	0.4135	6.3057
	α	1.5401	0.1401	0.0695	1.4383	0.0383	0.1391	1.3837	0.0163	0.7334	1.3676	0.0324	0.1852	1.3744	0.0256	0.2641
	λ	0.0303	0.0420	0.0089	0.1317	0.0147	0.0402	0.2378	0.1028	0.2232	0.1774	0.0604	0.0359	0.2257	0.1087	1.1197
n = 50	θ	1.2853	0.0153	0.0086	1.3213	0.0513	0.0279	1.3599	0.0899	0.2176	1.3127	0.0427	0.0309	1.3420	0.0720	1.0761
	β	1.1601	0.0399	0.0366	1.1825	0.0175	0.1378	1.3661	0.1661	1.5103	1.2650	0.0650	0.1321	1.3136	0.1136	0.2900
	*c*	1.4544	0.0044	0.0275	1.5086	0.0586	0.0880	1.5638	0.1138	0.3710	1.5307	0.0807	0.2350	1.6342	0.1842	7.0338
	α	1.5092	0.1092	0.0299	1.4693	0.0693	0.0684	1.3735	0.0265	0.1108	1.4093	0.0093	0.0539	1.3948	0.0052	0.0878
	λ	0.0151	0.0268	0.0032	0.1065	0.0105	0.0141	0.1755	0.0855	0.0291	0.1453	0.0283	0.0093	0.1565	0.0395	0.0192
n = 100	θ	1.2901	0.0201	0.0043	1.3020	0.0320	0.0084	1.3114	0.0414	0.0305	1.3024	0.0324	0.0122	1.2991	0.0291	0.0192
	β	1.1656	0.0344	0.0158	1.1521	0.0479	0.0610	1.2856	0.0856	0.2473	1.2326	0.0326	0.0602	1.2536	0.0536	0.1118
	*c*	1.4579	0.0079	0.0121	1.4930	0.0430	0.0544	1.5013	0.0513	0.0943	1.4849	0.0349	0.0469	1.5166	0.0666	0.0901
	α	1.4828	0.0828	0.0152	1.4629	0.0629	0.0369	1.4020	0.0202	0.0484	1.4203	0.0203	0.0263	1.4056	0.0056	0.0481
	λ	0.0049	0.0166	0.0012	0.1093	0.0077	0.0063	0.1485	0.0315	0.0082	0.1315	0.0145	0.0029	0.1449	0.0279	0.0074
n = 200	θ	1.2904	0.0204	0.0022	1.2983	0.0283	0.0048	1.2999	0.0299	0.0119	1.2938	0.0238	0.0062	1.2953	0.0253	0.0111
	β	1.1731	0.0269	0.0063	1.1713	0.0287	0.0285	1.2461	0.0461	0.0769	1.2153	0.0153	0.0291	1.2452	0.0452	0.0832
	*c*	1.4568	0.0068	0.0059	1.4752	0.0252	0.0271	1.4863	0.0363	0.0681	1.4765	0.0265	0.0304	1.4926	0.0426	0.0626
	α	1.4726	0.0726	0.0116	1.4639	0.0639	0.0237	1.4155	0.0155	0.0300	1.4288	0.0288	0.0137	1.4169	0.0169	0.0296
	λ	0.0006	0.0123	0.0007	0.1072	0.0098	0.0039	0.1401	0.0231	0.0046	0.1272	0.0102	0.0017	0.1385	0.0215	0.0043
n = 300	θ	1.2909	0.0209	0.0016	1.2976	0.0276	0.0033	1.2996	0.0296	0.0090	1.2944	0.0244	0.0035	1.2958	0.0258	0.0083
	β	1.1768	0.0232	0.0036	1.1674	0.0326	0.0184	1.2186	0.0186	0.0571	1.2020	0.0020	0.0189	1.2393	0.0393	0.0544
	*c*	1.4575	0.0075	0.0032	1.4709	0.0209	0.0195	1.4973	0.0473	0.0553	1.4805	0.0305	0.0239	1.4737	0.0237	0.0378
	α	1.4573	0.0573	0.0066	1.4568	0.0568	0.0149	1.4238	0.0238	0.0131	1.4302	0.0302	0.0066	1.4284	0.0284	0.0168
	λ	0.0033	0.0084	0.0003	0.1085	0.0085	0.0024	0.1347	0.0177	0.0027	0.1244	0.0074	0.0009	0.1311	0.0141	0.0022
n = 500	θ	1.2886	0.0186	0.0011	1.2919	0.0219	0.0023	1.3000	0.0300	0.0045	1.2930	0.0230	0.0019	1.2960	0.0260	0.0053
	β	1.1811	0.0189	0.0020	1.1822	0.0178	0.0129	1.2216	0.0216	0.0468	1.2009	0.0009	0.0085	1.2157	0.0157	0.0284
	*c*	1.4578	0.0078	0.0017	1.4548	0.0048	0.0143	1.4777	0.0277	0.0318	1.4709	0.0209	0.0097	1.4729	0.0229	0.0226

**Table 4:** Simulation study at α=26.4,λ=11.7,θ=12.77,β=15.2 and c=33.45.

Set IV:			MLE			PE			LSE			WLS			CVM	
		Est	Bias	MSE	Est	Bias	MSE	Est	Bias	MSE	Est	Bias	MSE	Est	Bias	MSE
	α	26.959	0.5589	7.5747	28.4682	2.0682	38.2159	27.7222	1.3222	12.0924	27.7749	1.3749	11.1449	27.185	0.7850	13.618
	λ	11.7008	0.0008	0.0000	11.7195	0.0195	0.3076	11.6983	0.0017	0.0005	11.6992	0.0008	0.0001	11.699	0.0010	0.0015
n = 20	θ	12.7682	0.0018	0.0001	12.8003	0.0303	0.3106	12.7749	0.0049	0.0008	12.7741	0.0041	0.0003	12.768	0.0020	0.0028
	β	15.7237	0.5237	6.1200	14.6361	0.5639	7.6170	14.7013	0.4987	8.4308	14.9269	0.2731	6.1488	15.6491	0.4491	10.1611
	*c*	34.5434	1.0934	9.5238	32.9924	0.4576	11.6788	34.3149	0.8649	10.0987	34.0997	0.6497	12.1072	34.8646	1.4136	14.6085
	α	27.5588	1.1588	4.1070	27.8862	1.4862	23.8686	27.6495	1.2495	7.0637	27.502	1.1020	5.4489	27.480	1.8066	6.2983
	λ	11.7001	0.0001	0.0000	11.7137	0.0137	0.1236	11.699	0.0010	0.0001	11.6988	0.0012	0.0001	11.7001	0.0001	0.0001
n = 50	θ	12.770	0.0000	0.0001	12.7907	0.0207	0.1239	12.772	0.0020	0.0001	12.7707	0.0007	0.0001	12.7702	0.0002	0.0002
	β	15.2537	0.0537	1.6628	14.7424	0.4576	3.3262	14.984	0.2160	2.6378	15.0114	0.1886	2.1777	15.2474	0.0474	2.6965
	*c*	34.1511	0.7011	3.1450	33.025	0.4250	6.9790	33.8796	0.4296	5.0640	34.0749	0.6249	3.4004	34.3073	0.8573	4.6293
	α	27.5869	1.1869	2.8986	27.8717	1.4717	17.2203	27.3975	0.9975	4.4564	27.3426	0.9426	4.3602	27.3833	0.9833	4.3350
	λ	11.700	0.0000	0.0000	11.7213	0.0213	0.0898	11.6998	0.0002	0.0001	11.7002	0.0002	0.0000	11.6998	0.0002	0.0001
n = 100	θ	12.7704	0.0004	0.0000	12.7962	0.0262	0.0906	12.7715	0.0015	0.0001	12.7708	0.0008	0.0001	12.7702	0.0002	0.0001
	β	15.1676	0.0324	0.8408	14.8779	0.3221	1.6165	15.0514	0.1486	1.1741	15.1632	0.0368	1.0074	15.2200	0.0200	1.1464
	*c*	33.9912	0.5412	1.4202	33.1592	0.2908	4.1905	33.7700	0.3200	2.1968	33.9130	0.4630	2.5914	33.9005	0.4505	2.4131
	α	27.5717	1.1717	2.3043	27.6267	1.2267	17.1422	27.3015	0.9015	3.1061	27.2515	0.8515	2.4380	27.2668	0.8668	2.7787
	λ	11.6999	0.0001	0.0000	11.7092	0.0092	0.1159	11.6998	0.0002	0.0000	11.6997	0.0003	0.0000	11.7000	0.0000	0.0000
n = 200	θ	12.7705	0.0005	0.0000	12.7823	0.0123	0.1160	12.7709	0.0009	0.0001	12.7706	0.0006	0.0000	12.7704	0.0004	0.0001
	β	15.1082	0.0918	0.4600	15.0021	0.1979	0.8621	15.1164	0.0836	0.6027	15.1321	0.0679	0.4377	15.2064	0.0064	0.5787
	*c*	33.9281	0.4781	0.8101	33.2922	0.1578	4.0075	33.6655	0.2155	1.3455	33.6704	0.2204	0.9582	33.7146	0.2646	1.2381
	α	27.5263	1.1263	2.0519	27.6812	1.2812	14.8407	27.1803	0.7803	2.3517	27.1481	0.7481	1.9804	27.1485	0.7485	2.3011
	λ	11.6999	0.0001	0.0000	11.7235	0.0235	0.1071	11.6998	0.0002	0.0000	11.7000	0.0000	0.0000	11.6999	0.0001	0.0000
n = 300	θ	12.7706	0.0006	0.0000	12.7962	0.0262	0.1081	12.7708	0.0008	0.0000	12.7707	0.0007	0.0000	12.7704	0.0004	0.0000
	β	15.0942	0.1058	0.3095	15.0614	0.1386	0.6665	15.1102	0.0898	0.4248	15.1295	0.0705	0.2934	15.1755	0.0245	0.4101
	*c*	33.8995	0.4496	0.5727	33.3532	0.0968	4.0181	33.6321	0.1821	0.9760	33.6270	0.1770	0.7044	33.6533	0.2033	0.9123
	α	27.4614	1.0614	1.6366	27.4341	1.0341	10.3563	27.0665	0.6665	1.5764	27.0010	0.6010	1.3168	27.0335	0.6335	1.5377
	λ	11.6998	0.0001	0.0000	11.7071	0.0071	0.0077	11.6998	0.0002	0.0000	11.6997	0.0003	0.0000	11.6999	0.0001	0.0000
n = 500	θ	12.7707	0.0007	0.0000	12.7792	0.0092	0.0078	12.7707	0.0007	0.0000	12.7704	0.0004	0.0000	12.7705	0.0005	0.0000
	β	15.0537	0.1463	0.2185	15.0945	0.1056	0.4263	15.1238	0.0762	0.2259	15.1508	0.0492	0.1933	15.1586	0.0414	0.2188
	*c*	33.8630	0.4130	0.4632	33.3099	0.1401	1.6474	33.5538	0.1038	0.5259	33.5366	0.0866	0.4235	33.5766	0.1266	0.4954

**Table 5:** Parameter estimates, SE in (), and confidence intervals’ length.

Data	$\hat{\alpha }$	$\hat{\lambda }$	$\hat{\theta }$	$\hat{\beta }$	$\hat{c}$
	0.0636	0.6008	1.4638	1.5414	2.0284
Failure Time Data	(0.2058)	(0.5712)	(1.6226)	(2.1527)	(2.8330)
	0.8069	2.2391	6.3607	8.4387	11.1052
	5.4361	−1.8154	3.1189	4.3767	0.4850
Gauge Lengths of 10 mm	(58.4270)	(0.0987)	(15.8010)	(32.2497)	(3.5736)
	229.0337	0.3870	61.9401	126.4189	14.0085
	2.0395	1.5950	3.4368	3.9508	3.0019
Strength Data	(24.6323)	(2.6199)	(4.3530)	(34.7129)	(26.3750)
	96.5587	10.2699	17.0638	136.0747	103.3899
	0.2493	−3.8664	7.1817	1.7261	0.6840
Student Grades Data	(5.3829)	(0.3589)	(131.3642)	(14.7891)	(5.8606)
	21.1010	1.4071	514.9477	57.9732	22.9736

**Table 6:** GOF criteria for the failure time data.

	−L	AIC	BIC	CAIC	HQIC	K-S	A-D	*p*-Value
NLW	127.4759	264.9517	277.1058	265.7209	269.8376	0.0831	0.5198	0.6079
Lomax	162.877	329.7540	334.6156	329.9021	331.7083	0.3028	11.5410	4.09$\;\times \;10^{-7}$
TWPL	136.3531	280.7062	290.4295	281.2126	284.6149	0.1061	1.4479	0.3004
OLIW	179.4022	366.8044	376.5277	367.3108	370.7131	0.3433	15.9210	5.054$\;\times \;10^{-9}$
EGMW	127.8997	265.7995	277.9536	266.5687	270.6853	0.0880	0.7010	0.5340

**Table 7:** GOF criteria for the gauge data.

	−L	AIC	BIC	CAIC	HQIC	K-S	A-D	*p*-Value
NLW	56.00961	122.0192	132.7349	123.0719	126.2338	0.0680	0.2568	0.9326
Lomax	133.4458	270.8915	275.1778	271.0915	272.5773	0.4862	18.8430	2.32$\;\times \;10^{-13}$
TWPL	59.5777	127.1554	135.7279	127.8451	130.5270	0.1023	0.6891	0.5250
OLIW	60.93501	129.8700	138.4426	130.5597	133.2416	0.0914	0.5359	0.6684
EGMW	59.27542	128.5508	139.2665	129.6035	132.7654	0.0935	0.5903	0.6406

**Table 8:** GOF criteria for strength data.

	−L	AIC	BIC	CAIC	HQIC	K-S	A-D	*p*-Value
NLW	14.28529	38.5706	49.2863	39.6232	42.7851	0.1342	0.9107	0.2063
Lomax	88.83032	181.6606	185.9469	181.8606	183.3465	0.4180	18.4240	5.513$\;\times \;10^{-10}$
TWPL	21.55742	51.1148	59.6874	51.8045	54.4865	0.2117	2.7566	0.0071
OLIW	47.15311	102.3062	110.8788	102.9959	105.6779	0.3589	10.3210	1.796$\;\times \;10^{-7}$
EGMW	14.92291	39.8458	50.5615	40.8985	44.0603	0.1407	0.9803	0.1648

**Table 9:** GOF criteria for student grades.

	−L	AIC	BIC	CAIC	HQIC	K-S	A-D	*p*-Value
NLW	195.4412	400.8825	410.2385	402.3111	404.4181	0.0842	0.3233	0.8854
Lomax	204.1959	412.3919	416.1343	412.6586	413.8061	0.2044	2.5909	0.0363
TWPL	201.681	411.3620	418.8468	412.2922	414.1905	0.1330	1.1214	0.3637
OLIW	215.6216	439.2432	446.7280	440.1734	442.0717	0.3132	5.8335	0.0002
EGMW	196.8086	403.6171	412.9731	405.0457	407.1528	0.0938	0.3520	0.7919

## Data Availability

Data is contained within the article.
